# Chronic long‐COVID syndrome: A protracted COVID‐19 illness with neurological dysfunctions

**DOI:** 10.1111/cns.13737

**Published:** 2021-10-09

**Authors:** Abdul Mannan Baig

**Affiliations:** ^1^ Department of Biological and Biomedical Sciences Aga Khan University Karachi Pakistan

**Keywords:** COVID‐19, long‐COVID, neurological deficits, SARS‐CoV‐2

## Abstract

After almost a year of COVID‐19, the chronic long‐COVID syndrome has been recognized as an entity in 2021. The patients with the long‐COVID are presenting with ominous neurological deficits that with time are becoming persistent and are causing disabilities in the affected individuals. The mechanisms underlying the neurological syndrome in long‐COVID have remained obscure and need to be actively researched to find a resolution for the patients with long‐COVID. Here, the factors like site of viral load, the differential immune response, neurodegenerative changes, and inflammation as possible causative factors are debated to understand and investigate the pathogenesis of neuro‐COVID in long‐COVID syndrome.

## Graphical abstract

The SARS‐CoV‐2 viral loads in nose are now known to reach the brain via cribriform plate. If the neurons in the CNS experience cellular degeneration due to SARS‐COV‐2, it is expected that patient with Long‐COVID syndrome would exhibit neurological deficits.

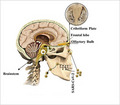

## 1 CHRONIC LONG‐COVID SYNDROME

Long‐COVID syndrome is a complex chronic clinical manifestation of signs and symptoms in patients who have experienced the SARS‐CoV‐2 infection without complete recovery from the COVID‐19.

A growing fraction of the COVID‐19 patients are emerging with continuing effects of the disease, with complaints like mental fog, delayed latent periods in recalling events of the recent past, tachycardia, and extreme fatigue.^1^ In the case where Long‐COVID syndrome is not observed after SARS‐CoV‐2 infections, patients have been reported to turn asymptomatic before or within 8–12 weeks after COVID‐19. There have been attempts to draw a timeline for Long‐COVID and clinical findings persisting beyond 3 weeks after the acute short phase was recommended to be considered for Long‐COVID syndrome^2^ though cases are appearing even 3 months after the acute phase of COVID‐19.

## 2 NEUROLOGICAL DYSFUNCTIONS IN LONG‐COVID

Infections caused by SARS‐CoV‐2 have not only resulted in a gigantic number of morbidities and mortalities worldwide in COVID‐19, but its ability to cause a protracted illness in form of long‐COVID is now becoming increasingly evident.^2^ The syndromic manifestation in long‐COVID has shown to become chronic and the affected individuals are seen to be predominately exhibiting neurological deficits which are worrying. The most daunting task in long‐COVID is to understand the mechanisms underlying mechanism in general and the pathogenesis of neurological signs and symptoms in particular. Debated here are local routes and systemic mechanisms by which SARS‐CoV‐2 can involve the nervous system to evoke a low‐grade smoldering neuronal injury that possibly contributes to the neurological deficits reported. A better understanding of the route and pathways of the SARS‐CoV‐2 in the causation of the neurological deficits is expected to develop targeted therapies in long‐COVID syndrome to minimize the disabilities resulting in the affected patients. Additionally, a history of pre‐COVID existing neurological deficit should be taken into account to identify newer neurological sign and symptoms caused by COVID. A differential diagnosis with post‐traumatic stress disorder (PTSD) caused by the pandemic appears to be important.

Also feature like facial drooping, and speech‐related deficits which are potential signs of stroke can be blunted by a patient's face mask, wearing of which is common during the COVID‐19 pandemic.^3^

## 3 SITES OF VIRAL LOAD AND THEIR SIGNIFICANCE IN NEURO‐COVID

**FIGURE 1:**
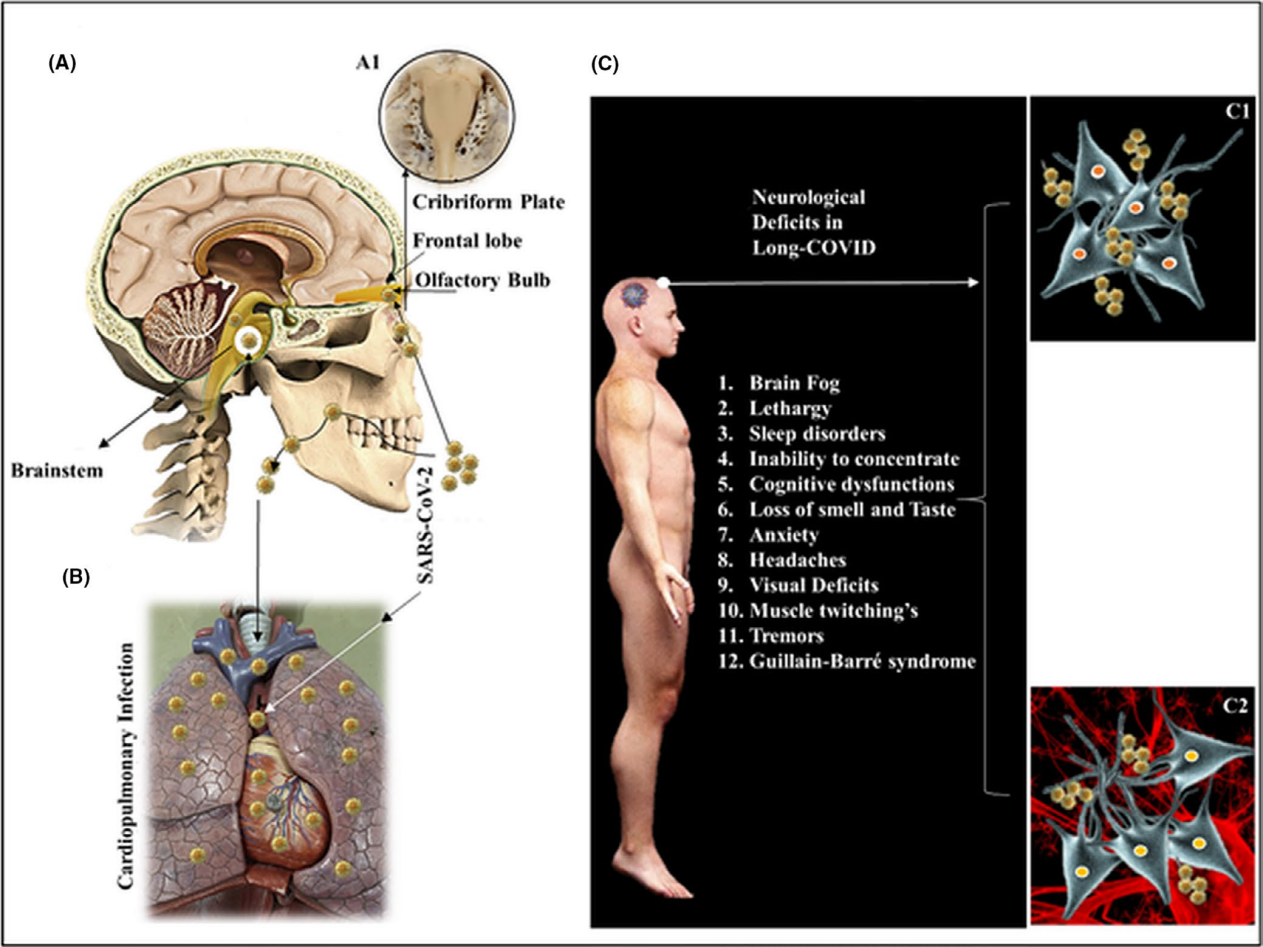
Long‐COVID: CNS Access and NeuroCOVID syndrome. SARS‐CoV‐2 viral loads can follow anatomical pathways from the nasal cavity to the brain (A‐A1) via the cribriform plate of the ethmoid bone (A1) and the lungs via respiratory passages (B). Patients in long‐COVID report diverse neurological signs and symptoms (C‐list) that can be due to slow neuronal damage (C1) (neurodegeneration) or due to low‐grade inflammation and cytokine‐mediated damage (C2). SARS‐CoV‐2 has been isolated from beneath the cribriform plate and the neurons of the frontal lobe in COVID‐19 patients

Central to the understanding of the neurological deficits caused by SARS‐CoV‐2 is the ability of this virus to seed specific sites in human tissues, which can enable its access to the central nervous system (CNS). Nasal and oral cavity are known sites of SARS‐CoV‐2 seeding from which samples are collected to diagnose COVID‐19. The nasal cavity, in particular, is anatomically extended to regions bearing neurological tissues like olfactory mucosa (OM), and across the cribriform plate, at the root of the nose (Figure 1A), to the olfactory bulb (OB) and frontal lobe of the brain (Figure 1A‐A1). Though the oral cavity has no direct extensions to the CNS, the infection of the nerve terminals in the mouth via retrograde neuronal transport can enable the SARS‐CoV‐2 to reach cardinal regions of the CNS like the brainstem (Figure 1A).^2^ Common to both of the above routes is its extension to the lungs (Figure 1B), which is a known organ to be involved in SARS‐CoV‐2 infection in acute as well as long‐COVID.

## 4 PROGRESSIVE NEUROTROPIC DEGENERATIVE EFFECTS OF SARS‐CoV‐2

One of the possible reasons for the observation of neurological deficits in long‐COVID^4^ (Figure 1C), is a possibly a slowly progressive degenerative effect of the SARS‐CoV‐2 following the neuronal entry. Such finding has been reported recently to affected patients and it should be no surprise to see the degenerative processes^5^ (Figure 1, C1), reflecting as neurological deficits. Though the underlying mechanisms leading to neuronal damage are yet to be completely understood, neuronal atrophy, hypometabolism, and defects induced in synaptic transmission can be the covert pathways induced by SARS‐CoV‐2 in long‐COVID to inflict neuronal damage.

## 5 POSSIBLE ROLE OF THE INFLAMMATORY RESPONSE TO SARS‐CoV‐2 INFECTION IN CAUSATION OF THE CNS MANIFESTATION IN LONG‐COVID

The immunologically mediated cytokine release by glia and lymphocytes at the site of viral load and infection may offer an additional if not the only reason behind protracted neurological signs and symptoms seen in long‐COVID. The inflammation (Figure 1, C2) around the OM, cribriform plate, and OB to the presence of SARS‐CoV‐2^6^ could become the basis of continuation of anosmia and hypogeusia in long‐COVID. Similar mechanisms involving the regions around the frontal lobe (Figure 1, A) (in proximity to the olfactory bulbs) with a sub‐clinical low‐grade inflammation can be the possible basis of “brain fog,” cognitive functional deficits, persistent headaches, and diverse neurological findings (Figure 1C) seen in long‐COVID.

## 6 DIFFERENTIAL SYNDROMIC MANIFESTATION IN NEURO‐COVID

The neurological deficits exhibited in COVID‐19 are known to differ widely among the patients infected with SARS‐CoV‐2. One example is loss of smell (anosmia) and altered sense of taste (hypogeusia), presence or absence of seizures, loss of consciousness, and difficulties with involuntary breathing mechanics, which vary in their intensity from patient to patient. Also, the fact that a significant number of patients never enter the long‐COVID phase needs to be investigated and understood in‐depth. The extent of the viral load and CNS entry along with differential immune response among the affected patients may explain these disparities observed and could be the basis of the protracted course of COVID‐19 in that becomes chronic to manifest as long‐COVID.^7^ Mechanisms in particular where an incomplete clearance of the SARS‐CoV‐2 is involved can linger on for months and its clearance might help as supported by the findings in 30 to 40% of those who get the vaccine have reported improvements to their symptoms. The fact that vaccination provokes the formation of antibodies and T‐killer cells, which may clear the residual SARS‐CoV‐2 from the body, appears to be one possible reason and an explanation for the improvements mentioned above in the neurological signs and symptoms of long‐COVID.

## 7 PERSISTENCE OF VIRAL LOADS AT IMMUNOLOGICALLY PRIVILEGED SITES

Among diverse reasons for incomplete clearance of SARS‐CoV‐2 from the human body by the immune system after 2–3 weeks of the COVID‐19 infection is the possible ability of the virus to reside at immunologically privileged sites, from where its arrival into the circulation can lead to a protracted course of illness leading to long‐COVID. The eyes, lacrimal sac,^8^ testes, and placenta are few examples where SARS‐CoV‐2 can conceal itself, but why this is not the case with patient's not exhibiting long‐COVID remains to be resolved.

## 8 DISCUSSION AND CONCLUSION

The magnitude with which the human immune system would respond to COVID‐19 differs from person to person for few apparent reasons, that includes, (a) the viral load, (b) the ACE2 expression levels in the tissues, and (c) the diversity of immune response.^9^ It appears that following an acute phase if the removal of SARS‐CoV‐2 is not substantial enough, the sub‐acute infection persists and becomes chronic to exhibit as long‐COVID. Of the sites that suffer the effects of the residual virus and the inflammation that accompanies it, the CNS is the most venerable tissue. The latter is because of the inability of the neurons to regenerate following damage caused by either the virus (Figure 1, C1) or cytokines generated by the low‐grade inflammation (Figure 1, C2). Other tissues that can regenerate following the cellular injury, do not dominate the symptomatic picture of long‐COVID as a partial restoration of function is likely following COVID‐19. The finding that vaccinations help some patients with long‐COVID,^7^ needs in‐depth research to find the support the vaccination offers to a chronic symptomatic long‐COVID patient at the pathogenetic level. Stimulation of a weakly provoked immune system to eradicate the virus seems to be one possibility, but other mechanisms may be at play that is yet to be elucidated in long‐COVID. The neurological deficits in long‐COVID^4^ were predicted to impose a huge burden on the healthcare profession and it now is proving itself a major cause of disability in the post‐acute‐COVID patient group.

## 9 FUTURE DIRECTIONS

Research that includes long‐COVID patients with neurological deficits for understanding the basic underlying pathogenesis of neuro‐COVID is needed and the NeuroCOVID project is one example.^4^ The biological fluids of these patients need to be fully analyzed for cytokines. Imaging the CNS to notice morphological changes induced in long‐COVID is also required as the images can be compared in the coming years to see if the findings progress or remain static. Clinical assessment and appropriate treatment regimens are also essentially needed as if not halted in time, the neurological deficits may become a permanent disability that would not revert.

## CONFLICT OF INTEREST

Author Abdul Mannan Baig declares that he has no conflict of interests.

## Data Availability

Not applicable.
